# VIM- and IMP-Type Metallo-β-lactamase–Producing *Pseudomonas* spp. and *Acinetobacter* spp. in Korean Hospitals

**DOI:** 10.3201/eid0907.030012

**Published:** 2003-07

**Authors:** Kyungwon Lee, Wee Gyo Lee, Young Uh, Gyoung Yim Ha, Jihyun Cho, Yunsop Chong, the Korean Nationwide Surveillance of Antimicrobial Resistance group

**Affiliations:** * Yonsei University College of Medicine, Seoul, Korea; † Ajou University School of Medicine, Suwon, Korea; ‡ Yonsei University Wonju College of Medicine, Wonju, Korea; § College of Medicine of Dongguk University, Kyongju, Korea; ¶ Wonkwang University College of Medicine, Iksan, Korea

**Keywords:** *Pseudomonas* spp., *Acinetobacter* spp., metallo-β-lactamase, VIM β-lactamase, IMP β-lactamase, Korea, dispatch

## Abstract

We determined the occurrence of acquired metallo-β-lactamase (MBL)–producing bacteria in Korean hospitals. Among the isolates nonsusceptible to imipenem that were collected from 28 hospitals from 2000 to 2001, 44 (11.4%) of 387 *Pseudomonas* spp. and 38 (14.2%) of 267 *Acinetobacter* spp. infections produced MBL and had alleles of *bla*_VIM-2_ or *bla*_IMP-1_. MBL-producing isolates were detected in 60.7% of the hospitals.

Carbapenems are often used as a last resort for treating serious infections attributable to multidrug-resistant gram-negative bacilli because these drugs are stable even to extended-spectrum and AmpC β-lactamases. However, gram-negative bacilli with acquired metallo-β-lactamase (MBL), IMP-1, emerged and spread during the early 1990s in Japan (*1*). IMP-1 and its variants were then detected in other countries (*2*).

Another type of acquired MBL, VIM-1, was first reported in *Pseudomonas aeruginosa* in Italy (*3*), followed by reports of VIM-2 in France and Greece. VIM-2 was detected in *P. aeruginosa* in a Korean hospital isolated as early as 1995 (*4*). The occurrence of the VIM enzyme has continued to evolve: VIM-3 was reported in Taiwan (*5*), and VIM-4 in the United States (*6*).

The *bla*_IMP_ and *bla*_VIM_ genes are horizontally transferable because they are inserted in integrins, and some of these integrons are located on conjugative plasmids (*7*). Because of its ability to spread, carbapenem resistance related to IMP and VIM β-lactamase production has become a serious concern (*8*). Laboratory personnel and physicians must consider the therapeutic and infection-control implications of not detecting carbapenemase-producing bacteria (*9*). A large number of VIM-2–producing *Pseudomonas* spp. have been detected in a Korean hospital since 1995 (*4*), but the occurrence of MBL-producing isolates has not been studied at other Korean hospitals, despite the high prevalence of carbapenem-resistant *P. aeruginosa* and *Acinetobacter* spp (*10*). The aim of our study was to determine the occurrence of acquired MBL-producing *P. aeruginosa* and *Acinetobacter* spp. among isolates collected by Korean Nationwide Surveillance of Antimicrobial Resistance group hospitals. The MBL types produced and the sources of the MBL-positive isolates were also investigated. In addition, pulsed-field gel electrophoresis (PFGE) patterns were compared to determine intra- and interhospital spread of resistant strains.

## The Study

Nonduplicate, imipenem-resistant isolates of 387 *Pseudomonas* spp. and 267 *Acinetobacter* spp. were collected from 2000 to 2001 from 28 hospitals in the Korean Nationwide Surveillance of Antimicrobial Resistance group hospitals located in six cities or provinces. The identification of the species and the imipenem susceptibility were confirmed at the coordinating laboratory by using conventional tests (*11*) or ATB 32 GN system (bioMerieux, Marcy-l’Etoile, France) and by using the disk diffusion test (*12*), respectively.

MBL production was screened by using the Hodge test and the imipenem-EDTA double disk synergy test (*13*). The *bla*_IMP-1_ and *bla*_VIM-2_ alleles were detected by polymerase chain reaction (PCR), and three of the positive isolates were confirmed by sequencing, as described previously (*4*). *Xba*I-digested genomic DNA of *P. aeruginosa* isolates was separated by PFGE using the CHEF-DR-II system (Bio-Rad Laboratories, Hercules, CA) (*4*). The pattern was analyzed visually and by using UVIBand and Map software (UVItec Ltd., Cambridge, UK).

Some of the *Pseudomonas* and *Acinetobacter* isolates collected were not fully resistant to imipenem but showed intermediate resistance when retested. Among the isolates not susceptible to imipenem, 44 (11.4%) of 387 *Pseudomonas* spp. (42 *P. aeruginosa* and 2 *P. putida*) and 38 (14.2%) of 267 *Acinetobacter* spp. were considered MBL producers on the basis of positive results by the Hodge test and imipenem-EDTA double disk synergy test (Table 1). MBL-producing *Pseudomonas* spp. and *Acinetobacter* spp. were detected in 11 (52.4%) of 21 and 10 (41.7%) of 24 hospitals that were located in four of five and five of six cities or provinces, respectively. We detected the *bla*_VIM_ allele by PCR from all 42 isolates of MBL-producing *P. aeruginosa* and 2 isolates of *P. putida*. The *bla*_VIM-2_ and *bla*_IMP-1_ alleles were detected in 27 (71.1%) and 11 (28.9%) of 38 *Acinetobacter* isolates, respectively (Table 2). Nucleotide sequencing for three representative PCR-positive isolates confirmed the presence of the *bla*_VIM-2_ gene in one isolate each of *P. aeruginosa* and *Acinetobacter* spp., and the *bla*_IMP-1_ gene in one isolate of *Acinetobacter* spp.

**Table 1:** Detection of metallo-β-lactamase–producing isolates among imipenem-nonsusceptible isolates of *Pseudomoas* spp. and *Acinetobacter* spp.

Organism	City/province	No. hospitals (%)		No. isolates (%)		
		Tested	Positive	Tested		Positive
*Pseudomonas* spp.	Seoul	11^a^	4 (36.4)	144	12 (8.3)	
	Kyungki	2	2 (100)	40	6 (15.0)	
	Kangwon	2	1 (50.0)	57	2 (3.5)	
	Chulla	4	4 (100)	108	24 (22.2)	
	Kyungsang	2	0 (0)	38	0 (0)	
	Total	21	11 (52.4)	387	44 (11.4)	
*Acinetobacter* spp.	Seoul	11^a^	4 (36.4)	107	12 (11.2)	
	Kyungki	3	0 (0)	29	0 (0)	
	Kangwon	3	2 (25.0)	41	8 (19.5)	
	Chulla	3	1 (12.5)	25	13 (52.0)	
	Kyungsang	2	1 (50.0)	53	1 (1.9)	
	Chungchung	2	2 (100)	12	4 (33.3)	
	Total	24	10 (41.7)	267	38 (14.2)	

^a^Four were tertiary-care hospitals.

**Table 2:** Detection of *bla*_VIM-2_ and *bla*_IMP-1_ allele from metallo-β-lactamase–producing *Pseudomonas* spp. and *Acinetobacter* spp. by polymerase chain reaction

Organism	No. isolates (%)		
	Tested	*bla*_VIM-2_ positive	*bla*_IMP-1_ positive
*Pseudomonas aeruginosa*	42	42 (100)	0 (0)
*P. putida*	2	2 (100)	0 (0)
*Acinetobacter* spp.	38	27 (71.1)	11 (28.9)
Total	82	71 (86.6)	11 (13.4)

The MBL-producing strains were isolated mainly from intensive-care unit patients (31.7%) and other inpatients (50.0%); five (6.1%) were from emergency service and other outpatients (Table 3). Overall, MBL-producing isolates were mainly obtained from specimens of sputum (50.0%) and urine (29.3%). However, the proportion of MBL-producing isolates was relatively higher among urine isolates: 17.3% for *Pseudomonas* spp. and 29.2% for *Acinetobacter* spp. We obtained one MBL-producing *Acinetobacter* isolate from each of the following specimen types: blood, spinal fluid, pleural fluid, and venous catheter tip (Table 4).

**Table 3:** *bla*_VIM-2_ and *bla*_IMP-1_ allele-positive *Pseudomonas* spp. and *Acinetobacter* spp. isolated by service

Organism	No. isolates (%)				
	Outpatient	Inpatient	Intensive-care unit	Others	Total
*Pseudomonas* spp.	3 (6.8)^a^	26 (59.1)	11 (25.0)	4 (9.1)	44 (100)
*Acinetobacter* spp.	2 (5.2)^b^	15 (39.5)	15 (39.5)	6 (15.8)	38 (100)
Total	5 (6.1)	41 (50.0)	26 (31.7)	10 (12.2)	82 (100)

^a^Two were emergency service patients, and one was a urology patient.

^b^One was an emergency service patient, and one was a pediatric patient.

**Table 4:** *bla*_VIM-2_ and *bla*_IMP-1_ allele-positive *Pseudomonas* spp. and *Acinetobacter* spp. isolated by source

Source	No. (%) of isolates with metallo-β-lactamase						% positive by source
	*Pseudomonas* spp.		*Acinetobacter* spp.		Total		
Tested	Positive	Tested	Positive	Tested	Positive
Sputum	200	22 (11.0)	143	19 (13.3)	343	41 (12.0)	50.0
Urine	98	17 (17.3)	24	7 (29.2)	122	24 (19.7)	29.3
Wound	49	2 (4.1)	71	7 (9.9)	120	9 (7.5)	10.9
Other^a^	18	3 (16.7)	29	5 (17.2)	47	8 (17.0)	9.8
Total	387	44 (11.4)	267	38 (14.2)	654	82 (12.5)	100

^a^Others included one *Acinetobacter* isolate from specimens from blood, spinal fluid, pleural fluid, and a venous catheter tip.

The PFGE of the *Xba*I-digested genomic DNA of 39 isolates of *P. aeruginosa* showed 22 patterns (data not shown). Six isolates from one hospital had an identical pattern. Thirteen isolates (33.3%) belonged to another identical pattern—six from one hospital, two from each of two hospitals, and one from each of three hospitals, which were located in a city and two provinces.

## Conclusions

In this study, >10% of all imipenem-nonsusceptible isolates of *Pseudomonas* spp. and *Acinetobacter* spp. were attributable to MBL production (Table 1), and these MBL-producing isolates were detected in 62.5% of the participating hospitals. Our finding indicates that MBL-producing *P. aeruginosa* is more prevalent in Korea than in other countries (*2*) and that MBL-producing *Acinetobacter* spp. is increasing. The percentage of hospitals with MBL-producing isolates might have been higher if a larger number of imipenem-nonsusceptible isolates had been collected for this study.

VIM-2 was the only type of acquired MBL identified initially in Korea. VIM-2–producing *P. aeruginosa* was isolated at almost the same time in Europe (*7*) and Korea (*4*). However, IMP-1–producing isolates were rare until 2000 in Korea. Only one and three IMP-1-positive *P. aeruginosa* and *Acinetobacter* spp., respectively, have been isolated at the reference laboratory (4, unpub. data). In our study, 11 (28.9%) of 38 MBL-positive isolates of *Acinetobacter* spp. were IMP producers (Table 2). This increase suggests the possible introduction of IMP-producing strains of *Acinetobacter* spp. from Japan, where 28 isolates of *bla*_IMP-1_-positive *Acinetobacter* *baumannii* were reported in a hospital as early as 1994 to 1996 (*14*).

Rasmussen and Bush (*15*) predicted that an increase of MBL-producing organisms was inevitable, given the more frequent use of carbapenems. Imipenem has been used for only 9 years in Korea, but the imipenem-resistance rate of *P. aeruginosa* has rapidly risen from 6% in 1996 to 19% in 2001. A study by the Korean Nationwide Surveillance of Antimicrobial Resistance group showed that the mean imipenem-resistance rates of *P. aeruginosa* in 1997 did not differ substantially depending on hospital size, (i.e., 17% in medium hospitals [<1,000 beds] and 18% in large hospitals [>1,000 beds]). The mean resistance rates to imipenem were not lower than those to ceftazidime in 2000, i.e., 21% versus 18% in large hospitals and 20% versus 19% in medium hospitals (data not shown).

*Acinetobacter* spp. are also common nosocomial pathogens with multidrug resistance. The imipenem resistance rate of this organism isolated in Korea was found to be much lower than that of *P. aeruginosa,* but its resistance rate rose from 4% in the first quarter to 20% in the third quarter of 2002 at the coordinating laboratory (data not shown).

In our study, MBL-producing *Pseudomonas* spp. and *Acinetobacter* spp. were isolated mainly from sputum and urine specimens, and most (81.7%) isolates were from inpatients and intensive-care unit patients. Therefore, proper treatment of respiratory secretions and urine from intensive-care unit patients is considered an important aspect of preventing the spread of MBL-producing organisms. The presence of *P. aeruginosa* isolates with identical PFGE patterns among those collected not only from certain hospitals but also from different hospitals suggests that clonal spread is at least a part of the cause of intra- and inter-hospital dissemination of MBL-producing isolates. The presence of VIM-2-producing *Serratia marcescens,* *Enterobacter cloacae*, and *Achromobacter xylosoxidans* subsp. *denitrificans* (unpub. data) in other hospitals also suggests horizontal transfer of the resistance determinants.

Cornaglia et al. reported that five of seven patients infected with MBL-producing *P. aeruginosa* died, although the cause of death was difficult to establish with certainty (*16*). Clinical studies on the infection are rare because isolation of MBL-producing gram-negative bacilli increased only recently. We anticipate difficulties in treating patients infected with MBL-producing gram-negative bacilli, which can hydrolyze, in vitro, all available β-lactams, except aztreonam for which clinical efficacy is unknown.

Our study indicates the urgent need for action to prevent further spread of MBL-producing organisms. Previous experiences with penicillin-nonsusceptible pneumococci, methicillin-resistant *Staphylococcus aureus*, vancomycin-resistant *Enterococcus faecium*, and extended-spectrum β-lactamase–producing *Klebsiella pneumoniae* indicate that once resistant bacteria can become widespread and cannot be controlled (*10*). Our first task is to detect MBL producers among clinical isolates (*9*). Although the National Committee for Clinical Laboratory Standards document (*12*) does not contain procedures for detection, simple procedures are available (*13*).

The prevalence of *bla*_VIM-2_ allele-positive *P. aeruginosa* and *bla*_IMP-1_ allele-positive *Acinetobacter* spp. is increasing possibly because of clonal and horizontal spread of the resistance determinant in Korean hospitals. Sputum and urine from inpatients and intensive-care unit patients were found to be the main sources of MBL-producing isolates. Laboratories not only in Korea but also in other countries with carbapenem-resistant organisms must be prepared to screen MBL-producing isolates to determine the clinical impact and prevent further spread of MBL-producing organisms.
